# Vegetation management for urban park visitors: a mixed methods approach in Portland, Oregon

**DOI:** 10.1002/eap.2079

**Published:** 2020-02-24

**Authors:** Michelle L. Talal, Mary V. Santelmann

**Affiliations:** ^1^ Environmental Sciences Graduate Program Oregon State University Corvallis Oregon 97331 USA; ^2^ College of Earth, Ocean, and Atmospheric Sciences Oregon State University Corvallis Oregon 97331 USA

**Keywords:** ecosystem services, interviews, management, qualitative research, urban parks, vegetation

## Abstract

Urban park managers are tasked with maintaining ecological function and quality of parks while also meeting visitor preferences. The purpose of this study was to better understand how managers currently manage vegetation in parks of Portland, Oregon. Twenty‐one urban park manager interviews were completed regarding 15 parks, which included natural‐passive use, recreational‐active use, and multi‐use park types. Responses were coded for themes and patterns of meaning. Mixed methods were used to evaluate the urban park manager interview data in the context of visitor interview and plant community composition data collected at the same parks. Nonmetric multidimensional scaling ordinations were used to identify urban park manager and visitor perspectives correlated with different park types and their vegetation. Across park types, managers discussed maintenance as a favorite aspect of plant management, while ecosystem management was often described by managers of natural‐passive use parks. Some managers indicated that they would make no changes to plant management, but the majority provided detailed recommendations such as enhancing maintenance, increasing staffing, adding plants, updating infrastructure, and improving plant species selection. There are opportunities to better meet the preferences of both managers and visitors by continuing to maintain large trees and trail/path vegetation for accessibility, removing invasive/harmful plants, and improving plant selection to include those that are heartier, more colorful, produce flowers, and are disease resistant, climate adapted, and provide habitat for a variety of species. While urban park managers discussed how they incorporated visitor preferences and accessibility in plant management, they also described limitations such as funding, staff resources, and undesirable visitor behaviors. Increased communication and collaboration among governmental agencies, non‐profit organizations, and community members, as well as continued investment in park management and interdisciplinary mixed methods research have the potential to enhance the many ecological and social benefits of urban parks.

## Introduction

Urban parks promote biodiversity conservation and a range of social, physical, and psychological benefits for visitors (Alvey 2006, Benedict and McMahon 2012, Lovell and Taylor 2013, Svendsen et al. 2016, Frumkin et al. 2017). Urban greenspaces can decrease crime (Bedimo‐Rung et al. 2005), and promote human–nature connections (Church 2018) and positive intercultural and multiethnic interactions (Peters 2010). Park managers are tasked with maintaining a balance between the quality of resources and visitor experiences (Budruk et al. 2003). A multifunctional approach in urban parks can help support plant and animal biodiversity conservation, air quality, landscape and habitat connectivity, access to greenspace, and community engagement (Lovell and Taylor 2013, Meerow and Newell 2017). However, budget cuts, high population densities in urban areas, and overuse of parks can make it difficult for park managers to achieve and maintain sustainable function and quality of urban parks (Baur et al. 2013a, b, Chan et al. 2015). Yet, there is great potential for urban green space management and design to balance ecological goals with aesthetic experiences and leisure activities for visitors (Gobster et al. 2007). More interdisciplinary research is needed that incorporates both manager and visitor perceptions to improve design and management of urban parks (Selge et al. 2011, Lovell and Taylor 2013, Nielsen et al. 2014, Muratet et al. 2015).

Management activities in urban parks and greenspaces can positively influence the vegetation composition and diversity, with associated benefits for wildlife and people. Preservation of large, remnant trees, increased understory vegetation with high species richness of native plants, and habitat connectivity can lead to increased species richness of birds and bats (White et al. 2005, Fontana et al. 2011, Barth et al. 2015, Threlfall et al. 2016). While the presence of high plant biodiversity is sometimes negatively related to visitor preferences due to a desire for open space (Qiu et al. 2013), human wellbeing in urban areas is often enhanced by biodiversity (Miller 2005, Sandifer et al. 2015, Botzat et al. 2016, Palliwoda et al. 2017). Management that promotes natural landscapes with a level of order and care are generally positively perceived by park visitors, and landscaping practices that provide openness can increase accessibility (Kaplan and Kaplan 1989, Kaplan et al. 1998).

Park managers striving to holistically manage vegetation for both humans and the environment often face limitations such as lack of funding, undesirable visitor behaviors (e.g., litter and graffiti), and knowledge gaps related to cultural ecosystem services (Millennium Ecosystem Assessment 2005, Baur et al. 2013a, b, Svendsen et al. 2016, Palliwoda et al. 2017). Cultural ecosystem services can be defined as the ways in which nature benefits humans in relation to their spiritual and religious values, aesthetic values, intellectual development, and recreation and ecotourism (Millennium Ecosystem Assessment 2005). Additionally, urban recreational areas are typically under more pressure than rural recreational areas because they tend to have year‐round usage, compact space with pollution concerns, and high demand from growing populations (Budruk et al. 2003, Chan et al. 2015).

Several previous studies in Portland, Oregon have explored urban park visitor experiences and provided a range of management strategies and planning recommendations (Baur et al. 2013a, b, Morzillo et al. 2016, Church 2018). However, more research is needed concerning park managers perspectives on park benefits and management. Park managers are familiar with park infrastructure, programming, general maintenance, design, visitor use, and vegetation management. They are also aware of management limitations and can communicate with funding bodies, taxpayers, and government agencies about how to better support parks (Moyle et al. 2014). The rich knowledge and experiences of managers can be used to increase information sharing among park managers, visitors, and researchers, and increase the many ecosystem services provided by urban parks (Benedict and McMahon 2012). Our focal research question is “How do park managers currently manage vegetation in urban parks to meet the preferences of urban park visitors in Portland, Oregon?” We specifically explore management perspectives and priorities as they relate to the vegetation and visitors of different park types (Talal 2019, Talal and Santelmann 2019).

## Methods

### Study area

Portland is the largest city in Oregon (376 km^2^). Its population of 650,000 people is approximately 50.5% female and 49.5% male, with 23% non‐white ethnic minorities and 48% of individuals ≥25 yr old having a least bachelor's degree (United States Census Bureau 2017; Appendix S1: Table S1). The city has long rainy winters and short dry summers with an average annual precipitation of approximately 85–140 cm, depending on elevation or proximity to hills (Christy et al. 2009). The diverse plant species of the region have ecological importance and a range of cultural uses (United States Soil Conservation Service 1983, Pojar and MacKinnon 2016). They include native, nonnative, and invasive species (Christy et al. 2009). Portland's urban parks include features such as wetlands, river floodplains, steep forested hillsides, and rolling hillslopes (City of Portland, Oregon 2018). They provide a variety of amenities such as trails, paths, playgrounds, dog parks, picnic tables, covered areas, sports fields, parking lots, boat ramps, recreational fountains, splash pads, skate plazas, and gardens. Many parks are managed by Portland Parks and Recreation, a City of Portland bureau that has a strategic plan to promote sustainable landscapes, recreational opportunities, and equitable access (Portland Parks and Recreation 2017). The parks are managed as developed parks and natural areas, with particular management responsibilities to Land Stewardship, Urban Forestry, and Turf and Irrigation divisions within Portland Parks and Recreation.

### Research design

The study used a mixed methods approach to analyze and integrate qualitative research (semi‐structured interviews, observations; Given 2008) with quantitative approaches (field data collection, statistical analysis; Johnson et al. 2007). Qualitative research was conducted by interviewing urban park employees of different management levels such as supervisors, horticulturalists, ecologists, and technicians. While this study was designed to capture many perspectives, it is not a complete survey of park managers in Portland, and may not be representative of all Portland park managers. The manager responses have the potential to reveal what they like or dislike about current vegetation management and limitations, as well as their perceptions of visitor interactions with plants, visitor feedback, and how plants are managed to meet visitor preferences. Qualitative data from manager interviews were then assessed in relation to the previously collected data on plant community composition and biodiversity (Talal and Santelmann 2019) and park visitor interview data (Talal 2019). Data on plant community composition and biodiversity patterns were collected in summer 2017 (Talal and Santelmann 2019; Data S1: Park Sample Units and Percent Cover, Plant Species Notes). The parks were revisited in summer 2018 to take photographs, and to observe and interview 43 park visitors about their experiences and vegetation perceptions (Talal 2019). Interview questions for park managers were formulated and used for park manager interviews in 2018–2019 (Appendix S2).

### Qualitative methods for park manager interviews

The study uses a phenomenological inquiry methodology, which is a method that questions the structure and essence of lived experience (Rossman and Rallis 2003) and is focused on learning about participant perceptions, comparing and contrasting these responses, and reflecting on meanings. The parks were selected using a stratified random sampling design from a list of parks within the Portland city boundary (City of Portland, Oregon 2018). This study used a park classification originating from King County, Washington (King County 2016) as adapted by Weems (2016). The three general park types based on use included: (1) recreational‐active use parks that are highly developed with facilities that promote active play such as play equipment and sports fields, (2) natural‐passive use parks that are generally more passively used with benches, viewing areas, and trails, but do not have formal fields for active play, and (3) multi‐use parks with a general mix of passive and active uses. An additional natural‐passive use park, Forest Park, was added to the initial set of randomly selected natural‐passive parks because it is an iconic park and the largest urban park within the city (Portland Parks and Recreation 2011). To have a balanced study design, an additional randomly selected recreational‐active use park and a multi‐use park were added, for a total of 15 parks with five parks of each type. While we acknowledge that no single park typology can capture all of the variation in landscape design, management, and visitor use, the typology we used allowed us to sample a relatively diverse range of urban parks within Portland (Table 1).

**Table 1:** Parks by type, area, and date of establishment

Park	Park type	Hectares	Establishment date
Big Four Corners Natural Area	N	59	2005
Forest Park	N	2,058	1947
George Himes Natural Area	N	13	1903
Oaks Bottom Wildlife Refuge	N	65	1959
Woods Memorial Natural Area	N	19	1988
Brentwood Park	R	5.7	1951
Jamison Square	R	0.4	2000
Khunamokwst Park	R	1	2009
North Swan Island Boat Ramp	R	0.7	1996
Wallace Park	R	2.2	1920
Columbia Park	M	14	1891
Ed Benedict Park	M	5.1	1988
Patton Square Park	M	0.5	1960
Plaza Blocks	M	0.7	1869
Rose City Park	M	3.4	1920

Note

Park types are N, natural‐passive use; M, multi‐use; and R, recreational‐active use.

We used a publicly available Portland Parks and Recreation contact list to identify park managers for each of the 15 parks in the study (Portland Parks and Recreation 2018). Some park managers contacted referred us to other senior‐level managers that were more familiar with each park. We interviewed a set of “senior‐level” managers (i.e., supervisors and senior natural resource ecologists) for 15 parks, including participants from the Land Stewardship, Urban Forestry, and Turf and Irrigation divisions within Portland Parks and Recreation. Two parks of each park type were randomly selected for interviews of six additional “on‐the‐ground” managers that visit and maintain the park landscape most frequently (i.e., horticulturalists, ecologists, botanic specialists, and park technicians). Some participants managed multiple parks within the study and so, for 21 total interviews, there were 15 unique participants.

Interviews with the park managers were conducted between 10 December 2018 and 11 March 2019. Thirteen interviews were conducted by telephone and eight were in‐person. After the initial introductions and completion of the informed consent process (Appendix S2), managers were asked a range of questions about plant management in the park. Although interviews were not audio‐recorded, the responses were validated by reading the written responses to participants and asking for any clarification. Several questions were asked during the interviews, however, the ones used to answer the research question for this study included:

1. What aspects, if any, do you like about how the plants are managed in this park?
2. What aspects, if any, would you like to change about the way plants are managed in this park?
3. Do you feel that this park is accessible to the needs of visitors?
4. In your opinion, how do park visitors interact with the plants in this park?
5. Have you received comments from park visitors about the plants in this park? If so, describe.
6. Specifically, how does park visitor experience influence vegetation choice and/or design in this park?
7. What are the factors, if any, that limit your ability to manage the park in the way you might prefer?
8. Background questions: How long have you worked at Portland Parks and Recreation, and how many years have you worked in your profession?

Interviews were transcribed into text documents for content analysis (Rossman and Rallis 2003), and interview responses were coded using QSR International Pty Ltd., Version 12 (Melbourne, Australia) NVivo 12 Plus software to look for potential patterns. The data were evaluated to identify themes and patterns of meaning that emerged, and then codes were reviewed and refined into a codebook. The coding was validated by randomly selecting and sharing 5% of the interviews and the codebook with two other researchers, comparing the coding results (code overlap of 73%), and calculating a kappa statistic in NVivo (mean kappa coefficient of 0.85 for all nodes). The codebook for the manager interviews is provided (Appendix S3). A narrative was also created to provide detail on themes, meanings, and supporting quotes provided by participants.

### Mixed methods

#### Data structure

The qualitative manager interview data were assessed in relation to the quantitative plant community composition (Talal and Santelmann 2019) and the qualitative park visitor data (Talal 2019) collected at the same 15 parks using multivariate ordination techniques. The plant community composition data structure included the species matrix (15 sample units × 189 species), consisting of presence–absence data for trees, saplings/shrubs, and vines averaged for the five 400‐m^2^ square plots within each of the 15 urban parks. The vegetation data were used as the main matrix in the ordination as in Talal and Santelmann (2019). For this study, the park visitor and park manager interview data were used as explanatory variables in the second matrix of the ordination.

The park visitor data included observation and semi‐structured interview responses for 43 park visitors in 15 different urban parks. Three visitors were interviewed in each park, with the exception of Big Four Corners Natural Area, where the only person encountered during the visit was interviewed. The park visitor response matrix (15 sample units × 26 variables) contained count measurements of visitor responses, including what they liked about the plants (e.g., trees, beauty, diversity) or would like to change about the plants (e.g., more color or flowers, no changes), and any barriers to park accessibility (e.g., proximity, water, bathrooms; Table 2). The park visitor response matrix included responses to three questions similar to those asked of park managers (questions 1–3), related to the visitor’ likes and dislikes of vegetation, and if the park was accessible to their needs.

**Table 2:** Park visitor interview response variables (V), including accessibility, what they liked or would like to change about the plants

Variable	Question topic
Trees (V)	likes
Size (V)	likes
Colors (V)	likes
Diversity (V)	likes
Maintenance (V)	likes
Shade (V)	likes
Beauty (V)	likes
Maturity (V)	likes
Grass (V)	likes
No preference (V)	likes
No changes to plants (V)	changes
Color, more (V)	changes
Flowers, more (V)	changes
Invasive, harmful plant removal (V)	changes
Middle growth, shrubs, more (V)	changes
Placement of plants, improve (V)	changes
Proximity (V)	access
General access (V)	access
Maintenance (V)	access
Trails/Paths (V)	access
Relaxation (V)	access
Water (recreational, drinking) (V)	access
Bathrooms (V)	access
Benches (V)	access
No bathrooms (V)	access
Not all trails/paths (V)	access

The park manager interview responses (21 interviews total for 15 parks) to three similar questions plus an additional question about limitations were summarized, but only included the results for commonly mentioned responses provided by at least 10% of the interviews. The park manager response matrix (15 sample units × 53 variables) included count measurements of responses to questions 1–7, for example, what managers liked or would like to change about the plant managements, perceptions of visitor accessibility, visitor comments about the vegetation, how park visitor experience influences vegetation choice and/or design, and any limitations to park management (Table 3).

**Table 3:** Park manager interview response variables (M), including what they liked or would like to change about plant management, visitor accessibility, how visitor perceptions influenced plant management, and limitations

Variable	Question topic
Color, seasonal (M)	likes
Design, layout (M)	likes
Different crews in parks dept (M)	likes
Ecosystem management (M)	likes
Habitat variation (M)	likes
Horticultural beds (M)	likes
Dislike plant management (M)	likes
Maintenance (M)	likes
Plant variety (M)	likes
Shade (M)	likes
Trees (M)	likes
Funding, budget, more (M)	changes
Infrastructure, update (M)	changes
Maintenance, improve (M)	changes
No changes (M)	changes
Plants, more (M)	changes
Species selection, improve (M)	changes
Staffing, more (M)	changes
Unfavorable visitor behaviors, decrease (M)	changes
ADA/wheelchair accessible (M)	access
Boat ramp (M)	access
Parking (M)	access
Trails/paths (M)	access
Playground (M)	access
Proximity/location (M)	access
General access (M)	access
Not all ADA (M)	access
Not all trails/paths (M)	access
Visitors comment—hazard vegetation (M)	visitor comments
Visitors comment—invasive plants (M)	visitor comments
Visitors comment—replacement request (M)	visitor comments
Visitors comment—like vegetation (M)	visitor comments
Do not receive visitor comments (M)	visitor comments
Aesthetics, improvements (M)	visitor influence plant mgmt
Balance ecological and human needs (M)	visitor influence plant mgmt
Design, changed (M)	visitor influence plant mgmt
Design, kept (M)	visitor influence plant mgmt
Eliminate hiding/camping places (M)	visitor influence plant mgmt
Hazard plant removal (M)	visitor influence plant mgmt
Hearty plants to withstand trampling (M)	visitor influence plant mgmt
Not much public/visitor input (M)	visitor influence plant mgmt
Open space for activities (M)	visitor influence plant mgmt
Plants to guide human traffic (M)	visitor influence plant mgmt
Public input during planning (M)	visitor influence plant mgmt
Trail access (M)	visitor influence plant mgmt
Coordination in parks dept, limitation (M)	limitations
Enforcement, limitation (M)	limitations
Equipment/Supplies, limitation (M)	limitations
Funding/budget, limitation (M)	limitations
Infrastructure, limitation (M)	limitations
Staff resources, limitation (M)	limitations
Unfavorable visitor behaviors, limitation (M)	limitations
Water use, limitation (M)	limitations

Note

Abbreviations are dept, department; mgmt, management.

#### Data analysis

The plant community composition data used in this study were presence‐absence data, in which all occurrences received equal weighting regardless of abundance. The park visitor and park manager response data were also transformed into presence–absence data to provide equal weighting of responses. The relationships between the sample units, park visitor responses, and park manager responses were then characterized with non‐metric multidimensional scaling (NMS) in PC‐ORD Version 7. The NMS of the sample units in species space was completed using the “autopilot mode,” slow and thorough speed, a random starting configuration, 200 runs with real data, Sørensen (Bray–Curtis) distance measure, and penalizing unequal ordination distance. The final solution for the NMS ordination of the parks in species space was determined by plotting final stress vs. the number of dimensions and choosing the number of axes beyond which stress reductions were small (McCune and Grace 2002). Overlays of the park visitor and park manager data as joint plots were examined to evaluate the direction and strength of relationships between explanatory variables and the vegetation in the different park types, as expressed along the ordination axes.

## Results

### Park manager qualitative results

Background information about the managers interviewed is presented in Table 4. Interview responses were analyzed based on park type (i.e., natural‐passive, recreational‐active, and multi‐use parks). The quotations throughout this section provide the managers’ detailed perspectives.

**Table 4:** Number of park visits, average number of years at Portland Parks and Recreation, and average number of years in profession for park managers and by park type

Park type	Median no. visits/year	Average no. years at Portland parks and recreation	Average no. years in profession
All park managers	24	12.7	22.5
Natural‐passive use	30	7.8	17.2
Recreational‐active use	12	14.1	24.4
Multi‐use	18	16.6	25.8

### Natural‐passive use parks

Seven semi‐structured interviews were conducted with four participants regarding the five natural‐passive use parks Table 1. Natural‐passive use parks tend to have benches, viewing areas, and trails for walking, hiking, and biking, but lack formal fields for active play (King County 2016, Weems 2016). These parks have habitats ranging from wetlands and river floodplains to forested hillslopes. All have trails, but some (e.g., Forest Park) have more extensive trail networks and signage than others (e.g., Big Four Corners Natural Area).

The managers gave a range of responses to questions concerning what they liked about the management of their natural‐passive use parks, such as focusing on ecosystem management for ecological functions and processes, maintenance (e.g., invasive species management), habitat variation, native species, plant variety, trails, trees, and understory, and the involvement of volunteers. The most common responses from participants were that they appreciated the ecosystem management approach and invasive species management in these parks:

“…this site is typical of most of our natural areas in that we are managing per those ecosystem management strategies I've laid out…We are managing on a landscape scale rather than as a horticulturist on a plant‐based basis. We don't manage for individual trees; we manage for habitats for ecological functions and processes.”

“…I like that we basically manage the park for natural succession. Because of its size and integrity, we are able to remove disturbances like invasive plants, namely ivy, blackberry, and clematis. And once we've removed those disturbances, we often see a rebounding from the native seed bank that has been suppressed in the soil.”

“…there are some areas in the park that are contiguous, but we can't work on them because they aren't ours. And so, the most invaded, weediest places are not City of Portland park property at this point. It would be nice to have some agreements with adjacent landowners to be able to manage. On one side, it's your first entry and it's a very weedy corridor. So, the perception is that in some of these sections is that the Parks Bureau is not taking of it.”

Another manager appreciated the variety of plants and habitat types, and how they enhanced recreational opportunities:

“… we have a cedar grove with older, really spectacular trees. And there's more variation in habitat types. Still forested, but you can transition from different places. The trail network—you can hike within the park and have a whole hiking experience. And there are some rare plants, or unusual anyway.”

The managers of natural‐passive use parks also described ways in which they would change plant management, which included additional staffing and funds, and reducing unfavorable visitor behaviors. Two managers indicated that they would make no changes to plant management. However, the most common response was to increase staffing and resources:

“We really could use more hands on the ground to do the work. We are very sensitive to the needs of the habitat in terms of the IPM (Integrated Pest Management Program). The types of treatments we are engaged in, we are doing well and I feel good about it. Portland Parks and Recreation does a good job of implementing this program on their sites.”

“I'd like to have enough resources to work on the park as a whole rather than having to do smaller projects over time as grant funds are available.”

While many managers indicated that staff and funding were what they would like to change about the plant management, they also described the challenges that some visitors created:

“There are people that will chop down trees (the homeless campers) for firewood, and there is compaction of the soil…as far as our practices, I wouldn't change anything.”

When the park managers were asked if they felt that the natural‐passive use parks in this study were accessible to the needs of park visitors, many of them noted that accessibility was complex and has several meanings, depending on the visitor. They discussed issues such as paths and trails, the American Disabilities Act (ADA), proximity and/or location, signage in different languages, a welcoming atmosphere, and access to wildlife and nature. Some managers acknowledged that trails and paths could be improved in terms of ADA accessibility and signage could be provided in languages besides English:

“…we have some room to improve in that area. The challenges that I identified (trailheads not being well marked or easy to find) make it difficult for all visitors to access…we only have one‐quarter of a mile that is ADA accessible. And most of our information and signage in the park is only English. Safety‐related signs are in English and Spanish, but permanent signs are only in English…we need to tap into technology a bit more. We've done outreach with specific groups and need to get more creative.”

Some managers of natural‐passive use parks focus more on managing habitat for wildlife:

“It's designed as wildlife habitat, so…the fact that there is a trail to walk along the outside of it, that provides some opportunities for wildlife viewing and enjoyment of the natural area. It's not designed to have a more developed trail system that other parks might provide.”

Managers of natural‐passive use parks indicated that visitors interacted with plants in a variety of ways, such as appreciating trees, colors, variety of plants, bird habitat, and flowers, followed by stewardship and volunteering, research and environmental education. They also noted hazards such as fallen trees or damaging activities by visitors (trampling, breaking, graffiti, picking flowers, and dogs running off‐leash.

“I would say [visitors interact with the plants] typically respectfully. We do have issues were people in wildflower season will want to pick wildflowers. We have a small group of people that engage in graffiti, which can include the trees. And the only major issue for us is dogs. It is not uncommon for people to bring their dogs to the park and have them off‐leash, which is against our rules. And those animals can impact the vegetation in the parks.”

All but two of the managers reported that they had received comments about the vegetation. The most common comments from visitors cited concerned management of hazard vegetation. They also noted comments of appreciation for plants or new plantings, and concern about plants that had been removed or graffiti on trees. Some managers felt that park visitors focused more on recreational concerns rather than plants.

In response to questions about how visitor experience influences vegetation choice and/or design in the parks, managers primarily discussed vegetation management for trail access and hazard plant removal, followed by improving aesthetics and eliminating hiding/camping places. Additional responses included balancing human and ecological needs, updating designs, adding plantings to hide adjacent residences, maintaining views, and involving community volunteers in plantings or removing invasive plants.

“[We] try to keep the vegetation attractive, non‐harmful (so we won't plant stinging nettle, for example). We need to be careful of what we call hidey‐holes, which areas of dense vegetation that people can hide…so, we try to group plants and leave spaces between the groups, so that you can see…we also try to disguise chronic camping areas and social trails. Sometimes it's planting those trail areas or putting down brush and large woody debris in those areas.”

“I look at what's missing in terms of upper canopy, midstory, and understory. So, we have a really good upper canopy. The midstory is lacking. The understory is mixed. So, when I choose midstory plants, I look at both the ecological benefit and the visual benefit. We look for things that have both. Like red elderberry is very good for wildlife and visually appealing for park users. So, I do try to have a diverse mix of plants that will ultimately make the site more interesting…like vine maple, a midstory that changes color in the fall. Indian plum is one of the first things to flower in the spring. So, we get to have both benefit and visual interest.”

While most managers discussed their own management efforts, others felt that volunteers and stewards had a large impact and that visitor experiences influence vegetation management:

“Quite a bit. We looked at converting what was a former ball field into a pollinator meadow. A legacy from when it was a Multnomah County park. And so, a lot of thought went into that conversion. We've been working on it for the last four years. It used to be overgrown with blackberries…we've also had a lot of planting events out there with Friend's Groups, and at the site in general.”

When asked about factors that limited their ability to manage the parks in the ways they might prefer, the most common responses were staff resources, funding, and unfavorable visitor behaviors (e.g., homelessness and camping, dogs off‐leash, trash). Less frequent responses included the need for additional scientific information and limitations associated with steep terrain, equipment, and/or supplies.

“If I had my way, we would have more resources to put into the work that we are doing. We are working hard to keep the native habitat native, and that's challenging with limited resources. I would funnel more resources to [a large natural‐passive use park]. It's a large acreage, provides a lot of diversity of ecological niches for different wildlife species. It's kind of special in that regard. It has access to water and there is a large constructed wetland on the site, and that provides special habitat opportunities for waterfowl and beaver…. Like all land managers, we have limited resources and a lot of land to manage. Resources in terms of people and equipment.”

“We always could use more staff and funding. Especially when it comes to our park rangers where they are there to dissuade negative behavior and to implement the rules and regulations of the park. We have a lot of people that break the rules and without the staffing, it's hard to implement those rules like dogs off leashes, camping, and trash.”

### Recreational‐active use parks

Seven semi‐structured interviews were conducted with seven participants regarding five recreational‐active use parks (Table 1). Recreational‐active use parks can be generally described as highly developed with formal facilities promoting active use such as play equipment and sports fields. They often lack abundant greenspace and natural amenities that are typically found in natural‐passive use or multi‐use parks (King County 2016, Weems 2016). These parks have different amenities such as playgrounds, dog parks, picnic tables, covered areas, sports fields (e.g., softball, soccer, basketball), large parking lots, a boat ramp, a recreational fountain, splash pads, and a skate plaza.

The managers of the recreational‐active use parks provided many reasons why they liked the plant management, such as their maintenance (e.g., weed/invasive management, good maintenance, pruning, low maintenance plants), design/layout, horticultural beds, naturalistic landscape, and management for trees, shade, and seasonal color.

“The weed management in the landscape, horticultural beds with bark, and the pruning of the plant material is good. The turf quality is generally good in that park, unless you go after a rainy weekend. The zone supervisor and the horticulturalist are doing a good job in this park.”

“…it's a lovely little park. If I lived in the area, it would become my park. I would take my hat my off to whoever designed it. I think this is one of the parks that thought of trees. They thought of the hot, summertime experience where you're going to need shade.”

Although many participants described positive attributes of the plant management within the recreational‐active use parks, some managers did not like the plant management in their parks:

“I don't like how the plants are managed in this park. There's very few established plants.”

“There's potential to increase its benefits. We have a good infrastructure with beds laid out around the perimeter and parts of the interior, so we already have areas that can accept improvements without having to convert other areas.”

“I don't think have enough labor power to manage them in the way I'd like them to be managed…[we] could use more pruning and fertilizing, like we do in the private industry.”

The managers of the recreational‐active use parks in described a range of ways that they would like to change park management such as improved maintenance (i.e., more fertilizer and pruning), better plant species selection (i.e., climate‐adapted, disease‐resistant, diversity, more herbaceous perennials for pollinators), infrastructure updates, and more plants (e.g., midstory/shrubs, understory). Less frequently discussed changes included creating a new design and increasing staff training for a newly installed ecoroof.

“I'm not down with all the species that were selected. They chose a lot of birch and maple trees. Species selection could have used some help…we would have chosen trees that #1: were less susceptible to pests and pathogens, and #2: trees that are better able to handle climate changes issues…living in the Northwest, we have such a palette to choose from. Birch bronze bore—we are losing a lot of trees to this…maples—we have overabundance of maples in the City of Portland. And if we ever do get an infestation of Asian longhorn beetle (ALB), it's going to wipe out all the maples—and there are 12 species it likes. There are so many other trees we could have chosen. People go to it probably because it's beautiful in the fall, among other reasons.”

When recreational‐active use park managers were asked whether the parks were accessible to the needs of park visitors, all of them indicated that the parks were accessible, referring to access to paths and trails, ADA accessibility, and parking and boat ramp access. Some less frequently discussed accessibility features included cleanliness, open design, grass/turf, playground, proximity/location. Some participants discussed the potential to improve ADA accessibility, while others praised the ADA accessibility of the parks:

“Yes, it's accessible in that it has a nice, open design so that people can see the park from both ends and see across it, so it's inviting in that sense…good use of the space for getting around the park on those hard surfaces. It's successful to those with disabilities and children who would like to play in the playground, they can access it. Even the upper portion of nature play area has an access ramp and a transfer for that purpose.”

In response to questions about visitor interactions with the plants in the recreational‐active use parks, managers most often mentioned viewing interactions (i.e., for their beauty, seasonality, color, and shade) and unfavorable visitor behaviors (i.e., trampling/breaking vegetation, camping/hiding in shrubs, and garbage). Less frequent examples included walking around plants, playing in plants, obtaining shade, and using turf/grass for sitting or playing sports.

“I'd say, for the turf, the sports user groups are playing on the fields. I'd say families are picnicking on the grass. Other park users as they wander through the pathways are looking at the plants. They aren't necessarily interacting with them, but they are looking at the plants as the seasons change and the characteristics of those plants change through the seasons. I would say that there's usually a lot of people in the parks looking at the fall leaf color.”

While many participants described positive interactions between park visitors and the vegetation, they also explained ways that unfavorable visitor behaviors led to damaged plants:

“They throw garbage on them and trample them…I hate to say it, but there is not much interaction with the plants except to use it as screening material for undesirable behaviors. Not a lot of care is given to established plants by visitors.”

Six of the seven managers of recreational‐active use parks indicated that they had not received any comments from visitors, but one manager did report receiving positive feedback:

“It's looking good, good job. Thanks for your work!”

The managers described a range of ways that park visitor experience influences vegetation choice and/or design in the recreational‐active use parks. They primarily discussed keeping to the park design, improving aesthetics, eliminating hiding/camping places, and planting hearty vegetation to withstand trampling, and balancing human and ecological needs. Other responses included changing the park design, removing hazardous plants, creating open space for activities, using plants to guide human traffic, and soliciting public input during the planning process. Many managers felt that keeping to the original design was important for maintaining positive visitor experiences in the parks:

“…whoever designed this park, especially the trees along the street that create the allee (a design term). This design is incredibly intentional—it keeps people moving and makes the experience pleasant…they really did use trees as their element of design. I'm curious who designed it…”

While many managers described their intentions to keep to the original design, a manager discussed the benefits of some changes that were occurring in parks. They described how:

“We are going down the path of ‘Ecologically Sustainable Landscape Initiative’ for sustainability. We've heard that from park users that they would like to have more access to nature areas in the city limits. I don't know if they are planning on that in this park. They were starting out with just 10, but I don't know if it's one of the 10, and I don't know if there are any plans there.”

Some park managers also discussed how hazard vegetation removal and maintenance are important aspects of ensuring public safety in urban parks:

“…things just need to be low and hearty. Yeah, because you don't want to create hide‐y holes for people to hide in or do bad things.”

“Well in relationship to the trees, whenever there is a strong urban setting such as this park and there are trees planted, the maintenance of the trees is important…we think about how targets such as people or building could get hurt or damaged.”

When asked about the factors that limited their ability to manage the parks in the ways they might prefer, six out of seven participants indicated that funding was the primary limitation. Other, less frequently mentioned limitations included enforcement, staff resources, homelessness and trash, and water use. Managers described how a limited budget results in less irrigation, plantings, supplies, and the inability to enforce rules:

“One thing that we talk about a lot as people who have my position [is that] we could use a better plant budget to achieve these goals. A plant budget or funding source. Right now, most of our funding for these plants comes from the City of Portland's general fund, and that is not a secure source of funding. Our plant budget is very limited… It's also related to our budget for irrigation, which can be very important for getting them established and maintaining their health, but we are also under budgetary constraint on the water budget.”

### Multi‐use parks

During this study, seven semi‐structured interviews were conducted with six participants regarding five multi‐use parks (Table 1). Multi‐use parks are combinations of both recreational‐active use and natural‐passive use parks, and tend to promote both active and passive uses. For example, Ed Benedict Park has formal facilities for play equipment and sports, but also a memory garden, which promotes passive uses such as walking or sitting on benches and observing the flowers. Within these parks, there are different active use amenities such as playgrounds, a skate plaza, and sports fields (e.g., soccer, softball, and tennis). However, they each promote passive uses in a variety of ways such as having unpaved or paved trails and walking paths, a memory garden, and/or abundance of large and established vegetation cover.

When asked about what they liked about plant management in the multi‐use parks, some managers discussed their maintenance (i.e., well‐maintained, irrigation, low maintenance plants) and benefits of management by different work crews within Portland Parks and Recreation. Other attributes that they liked about the plant management included their beauty, seasonal colors, design/layout, grass, maturity, plant variety, and shade. Some managers discussed their appreciation of the maintenance and management of vegetation types by different work crews, while one manager indicated that the park design was somewhat problematic.

“I do like how we have different crews throughout our Bureau that manage each type of vegetation. So, we have an Urban Forestry crew that manages the trees and the canopy throughout the entire system. We have a Turf Crew that manages the sports fields. Individual zones, including our… park zone, manages understory vegetation, landscape beds and such. Because of that, all of those different types of vegetation get the kinds of needs they require based on that specialized work and based on their own allocation of resources…the whole park looks good as a whole, mainly due to the specialized workforces managing it.”

“I would say that my staff does the best they can. There are limited plant assets there. I don't really like the plant assets there that much. I think the design is somewhat problematic. I like the idea of shady, yet grassy areas for people to congregate. Conceptually, it is good.”

While some managers discussed the positive aspects of maintenance by different crews, others appreciated the landscape design and layout of the parks they manage:

“We have a vast variety of horticultural interest for year‐round. Things that are blooming in the early spring, summer, and fall. We have the beautiful old elm trees in the fall, that are stunningly beautiful.”

The managers noted a variety of ways that they would like to change plant management within the multi‐use parks, which included their maintenance (i.e., more pruning and watering), updating infrastructure, planting more trees and drought‐tolerant species, increasing staffing, improving plant placement, and reducing unfavorable visitor behaviors.

“We went through budget cuts this fiscal year and had to greatly reduce our watering schedules for pretty much all vegetation, including passive turf areas, landscape beds, and passive watering of trees (not including, the two‐year establishment for watering trees). Many of our mature trees get water from overspray of the turf. We didn't reduce watering for sports turfs because we permit them and we need them to be safe. The passive areas within trees were cut back about 10–20% and it makes a big difference especially when we need it most during the hot summer months.”

While most managers indicated aspects that they would like to change about management in the parks, a manager described how plant decisions from the past produced sustainable results.

“I wouldn't change anything, because…about 10 years ago, we put in plants for sustainability and for low water use. We started doing that long before we were asked to do it. We were definitely proactive on the sustainability factor.”

Many of the multi‐use park managers indicated that accessibility can have many meanings depending on visitors. However, they all felt that their parks were accessible because of their ADA accessibility (i.e., paths and a swing on a playground), parking, playground, proximity/location, park size, and drinking water.

“[The park] has good access system in it. We've recently improved the ADA access, which is important. It has an off‐street parking lot, which is helpful. And then it's a fairly high‐density neighborhood, so we get a lot of walk‐ins.”

When the multi‐use park managers were asked about how the visitors interacted with plants in the parks, they described viewing the trees, garden, appreciating their beauty, followed by obtaining shade and using the grass/turf. Most managers thought that the interaction between visitors and the plants was generally respectful:

“I think people are generally appreciative of the variety and types of plants, and they are complimentary and they are generally respectful of plantings in this park.”

However, one manager indicated that the plants were often trampled by visitors:

“…there's not a lot of respect for plant materials oftentimes and that is impacted also by design, or bad design. And you don't want somebody on a path and then have something like turf they want to access, and there is a planting pad in‐between, because they will just cut through. The other thing would be that people are not respectful of the ropes that we put up as we try to reestablish turf in the spring. It works for some people, while others don't care.”

Six of the seven managers indicated that they had received comments from park visitors about the plants. The most frequent comments were that the visitors liked the plants or had replacement requests for dead for dying vegetation, followed by wanting more shrub diversity.

“…if you are actively working on planting bed, then people will often stop to ask question or give feedback (laughs), and are often complimentary and appreciate: “This is so cool! I remember when this was…!”

Some managers were asked to remove dead or broken material, noting that volunteers were also helpful in removing invasive plant species:

“[Some ask] about some of the blackberry removal around the stairs, and so we took care of that. Some volunteers have also continued to maintain that, and that's about the extent regarding that request.”

The managers explained the many ways that park visitor experience influences vegetation choice and/or design in the multi‐use parks. They discussed the importance of planting hearty plants to withstand trampling, eliminating camping/hiding places, and using plants as guides for human traffic. Less frequent responses include keeping the original design, open space for activities, adding plants to popular areas, and soliciting public input in the planning process.

“Well, we have to put something: #1 – Sustainable. #2 – We have to design it in ways that people don't walk through the shrubs. We have to make sure things are low enough that people don't camp, because we have a large homeless population. It makes things safe. We don't want anything to be too high or too overgrown. Like a tall shrub that someone could hide in. We want it to be safe for people to walk through, night or day.”

Besides reducing the number of camping/hiding places and plant trampling, a manager described how it is important to think of visitor uses in parks to inform plant management:

“How should or how has it? We do consider what will be the primary activities in the park and primary usage, so planting is more limited if the desire is to have more open space for people to enjoy, especially in a smaller park. You don't want to lose square footage…”

In response to questions about the factors that limited their ability to manage the multi‐use parks in the ways they might prefer, five of the managers stated that funding was the primary limitation, others noted lack of staff resources, infrastructure, and coordination between work crews in Portland Parks and Recreation. Less frequently, they discussed the design plan, equipment/supplies, heavy usage/crowding, and tree canopy that shades grass.

I would say [the limitations] are financial. Another one would be the availability of other work groups to do major renovations, for example, the lawn. It needs regular aeration and fertilizer and top dressing (i.e., adding seed in the spring). I know those things may or may not happen because our turf maintenance facility is pretty stretched thin. They have a lot of parks and not very many people. They only do sport fields that get rented. If there are parks that aren't rented, then the turfs won't be maintained. They will prioritize the ones that will be leased, but I wish they would maintain all equally. It seems like the right thing to do. If you're going to have turf, then I think it should be maintained. Also, another factor are drainage improvements—they are expensive, but if we could do them, it would great.”

“…Turf, plumbing, electrical, irrigation, forestry, are all managed by different people, so I have no control of what work they do, when it will happen, if it will happen because they are managed by so many departments, it's very difficult to coordinate different needs…”

In addition to funding concerns, a manager found that staying consistent with the original design was a constraint, but that they still wanted to uphold the historical integrity of the park:

“Well, always funding would be one [limitation]. I think…the only limitation would be you'd have to stick with something traditional because of the history of the park. You couldn't do something sexy or tropical, you'd have to stick to the traditional nature for the park. If you have enough money and labor, you can do almost anything, but obviously we couldn't put a skatepark in there or anything. It would have to remain historical to the park.”

### Summary of park manager qualitative results

The previous sections provided detailed responses from 21 interviews about 15 urban parks for what managers liked or would like to change about how plants are managed, their perceptions of visitor accessibility, visitor interactions with the plants, and visitor comments. The managers also provided responses on how visitor perceptions of the plants influenced plant choice and/or design, as well as any limitations the managers have in managing the parks in the ways they might prefer. To visualize these perspectives, the count data for the interview questions (Data S1: Manager Responses—All Interviews, Manager Responses at Least 10% interviews, Vegetation Likes—Visitors and Managers, Vegetation Dislikes—Visitors and Managers, Accessibility—Visitors and Managers, How Visitors Influence Plant Mgmt, Comments from Visitors to Managers and Manager Limitations) and figures are provided for commonly mentioned responses in at least approximately 10% of interviews (Appendix S4: Figs. S1–S6).

### Mixed methods ordination results

**Figure 1:**
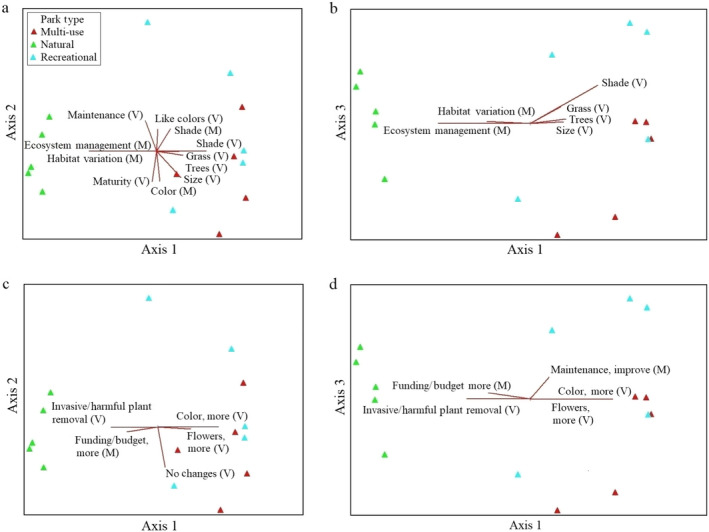
Nonmetric multidimensional scaling (NMS) ordinations for the parks in species space with variables that are strongly related to the individual ordination axes (*r* ^2^ > 0.2) shown as joint plots (radiated red lines with Visitor [V] and Manager [M] variables): (a) Axes 1 and 2 for “Likes” variables, (b) Axes 1 and 3 for “Likes” variables, (c) Axes 1 and 2 for “Changes” variables, and (d) Axes 1 and 3 for “Changes” variables.

**Figure 2:**
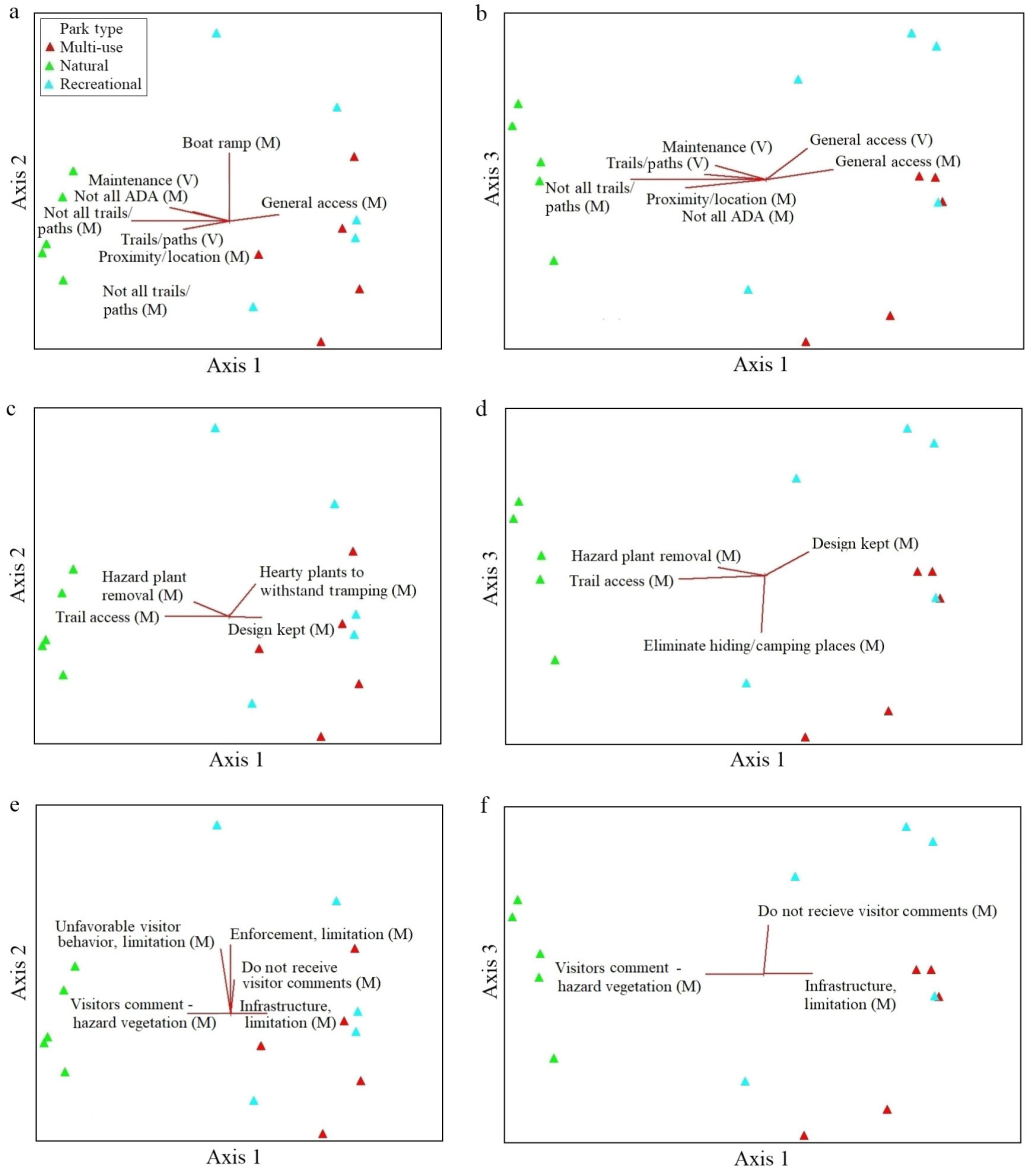
NMS ordinations for the parks in species space with variables that are strongly related to the individual ordination axes (*r* ^2^ > 0.2) shown as joint plots (radiated red lines with Visitor [V] and Manager [M] variables): (a) Axes 1 and 2 for “Access” variables, (b) Axes 1 and 3 for “Access” variables, (c) Axes 1 and 2 for “Visitor Perception Influences Plant Mgmt” variables, (d) Axes 1 and 3 for “Visitor Perception Influences Plant Mgmt” variables, (e) Axes 1 and 2 for “Comments” and “Limitations” variables, and (f) Axes 1 and 3 for “Comments” and “Limitations” variables. Note that mgmt stands for management.

The NMS ordination shows the parks (sample units) as triangles in species space with overlays of the variables as joint plots to reflect the direction and strength of linear relationships with the axes (Figs. 1, 2). The final solution for the NMS of the parks as sample units in species space was determined by plotting final stress vs. the number of dimensions and choosing the number of axes beyond which stress reductions were small (McCune and Grace 2002). A three‐dimensional solution was best for this data set with a final stress of 5.0 and a *R* ^2^_n_ (nonmetric fit) of 0.99. In NMS, a final stress of 5.0 is generally considered to be “good” (McCune and Grace 2002). Most of the variation is shown in the first two axes (*r* ^2^ = 0.82), with a total of *r* ^2^ = 0.92 for all three axes. Figs. 1a–d, 2a–f show the NMS ordinations, and Table 5 shows the strongest Pearson correlations for interview responses.

**Table 5:** Nonmetric multidimensional scaling (NMS) ordination's most strongly correlated Pearson correlations for interview questions

Question	Axis 1 Pearson correlations	Axis 2 Pearson correlations	Axis 3 Pearson correlations
What is liked about park vegetation?	“Ecosystem management (M)” (*r* = −0.79),“Shade (V)” (*r* = 0.68),“Habitat variation (M)” (*r* = −0.54)	“Maintenance (V)” (*r* = 0.53),“Maturity (V)” (*r* = −0.53),“Color, seasonal (M)” (*r* = −0.54)	“Shade (V)” (*r* = 0.51)
What would like to change about park vegetation?	“Color, more (V)” (*r* = 0.75),“Invasive, harmful plant removal (V)” (*r* = −0.66),“Flowers, more (V)” (*r* = 0.55), and “Funding/Budget, more (M)” (*r* = −0.53)	“No changes (V)” (*r* = −0.61)	“No changes (M)” (*r* = 0.42)
Is park accessible?	“Not all trails/paths (M)” (*r* = −0.79),“Not all ADA (M)” (*r* = −0.61),“General access (M)” (*r* = 0.56),“Trails/Paths (V)” (*r* = 0.54)	“Boat ramp (M)” (*r* = 0.66)	“General access (V)” (*r* = 0.38)
How does visitor perception influence plant management?	“Trail access (M)” (*r* = −0.64),“Hazard plant removal (M)”,“Design kept (M)” (*r* = 0.46).	“Hearty plants to withstand trampling (M)”	“Eliminate hiding/camping places (M)” (*r* = −0.52)
Comments that managers received from visitors and limitations to plant management?	“Visitors comment—Hazard vegetation (M)” (*r* = −0.53),“Infrastructure, limitation (M)” (*r* = 0.48)	“Enforcement, limitation (M)” (*r* = 0.66),“Unfavorable visitor behavior, limitation (M)” (*r* = 0.64)	“Do not receive visitor comments (M)” (*r* = 0.48)

Note

M, manager; V, visitor.

The final NMS solution with “likes” variables (visitor [V] and manager [M]) as joint plots are provided in Fig. 1a, b. Variables most strongly correlated with Axis 1 relate to managers’ appreciation of ecosystem management and habitat variation especially within the natural‐passive use parks, while visitors like the shade recreational‐active use and multi‐use parks. The variables most associated with Axis 2 are that visitors like the maintenance and maturity of the plants, while the managers like the seasonal color. For Axis 3, the most strongly correlated variable is that visitors appreciate the shade.

The final NMS solution with “changes” variables (visitor [V] and manager [M]) as joint plots are provided in Fig. 1c, d. The variables most strongly correlated with Axis 1 are related to the visitors’ preference for more color and flowers in the recreational‐active use and multi‐use parks and more invasive/harmful plant removal in the natural‐passive use parks, while the managers want more funding and budget for the natural‐passive use parks. The most strongly correlated variable with Axis 2 is that no changes are requested by visitors, and for Axis 3, the variable that no changes are requested by managers.

The final NMS solution with the “access” variables (visitor [V] and manager [M]) as joint plots are shown in Fig. 2a, b. The variables most strongly correlated with Axis 1 are that managers indicate that not all paths/trails are accessible and parks are not ADA accessible with regards to natural‐passive use parks. However, the managers indicate that the recreational‐active use and multi‐use parks are generally accessible, and visitors indicate that the trails/paths are accessible in natural‐passive use parks. The most strongly correlated “access” variable for Axis 2 is that the park managers discuss the presence of a boat ramp, and for Axis 3, visitors believe the parks are generally accessible.

The final NMS solution with “Visitor Perception Influences Plant Mgmt” variables (Manager [M]; mgmt stands for management) as joint plots are provided in Fig. 2c, d. Variables most strongly correlated with Axis 1 are managers believe that visitor perception influences park management in that there is trail access and hazard plant removal in natural‐passive use parks, and maintenance of park design in recreational‐active use and multi‐use parks. The most strongly correlated variable with Axis 2 is that the managers plant hearty plants to withstand trampling, and then for Axis 3, that the managers eliminate hiding/camping places.

The final NMS solution with “Comments” and “Limitations” variables as joint plots are provided in Fig. 2e, f. Variables most strongly correlated with Axis 1 are that managers receive comments from visitors about hazard vegetation for the natural‐passive use parks and managers are limited by infrastructure in the recreational‐active use and multi‐use parks. The variables most strongly correlated with Axis 2 are that managers have limitations related to enforcement and unfavorable visitor behaviors in primarily recreational‐active use parks. For Axis 3, the most strongly correlated variable was that managers do not receive visitor comments for primarily recreational‐active use parks. Variables related to increasing funding/budget and staffing do not appear on the ordination as highly coordinated with any of the axes likely because they were commonly mentioned by managers of all park types.

### Study limitations

The qualitative portion of this study included 21 semi‐structured interviews with managers of 15 urban parks interspersed throughout Portland, Oregon. While the study described their perspectives on plant management within parks, the study may not be representative of all park manager perspectives within the city. Additionally, there may be topics that were important to managers that were not discussed during the interviews. The NMS ordinations are useful for understanding some key aspects of manager and visitor perspectives in regards to the urban park plant community composition data, but should only be examined as a supplement to the detailed responses provided by participants during the interviews. Future studies of park managers could include a greater number and diversity of participants, as well as additional questions about what park managers like or dislike about vegetation beyond their management. The results of the manager interviews in this study, and in the study of visitor perceptions (Talal 2019) could also be useful in guiding preparation of more structured quantitative surveys.

## Discussion

Interdisciplinary research that incorporates both manager and visitor perceptions has the potential to improve habitat design and management approaches in urban parks (Lovell and Taylor 2013, Nielsen et al. 2014, Muratet et al. 2015). This study was unique in that it combined qualitative semi‐structured interviews with park managers and visitors at a range of urban park types interspersed throughout Portland, Oregon, and then evaluated these results alongside quantitative plant community composition data collected at these parks. The data were evaluated using NMS ordinations to determine the manager and visitor perspectives of plant management most strongly correlated with the different types of urban parks and their vegetation. The interviews with the park managers provided insights into ways that park managers currently manage vegetation in urban parks of Portland, Oregon to meet visitor preferences. The open‐ended nature of the questions also allowed the managers to share their own ideas about what they liked or would like to change about plant management, visitor accessibility, visitor interactions with plants, visitor comments, how visitors influence plant choice and/or design, as well as any limitations they may face in managing the parks in the ways they might prefer. By evaluating this information in relation to visitor preferences, there is a potential to increase information sharing among park managers, visitors, and researchers, and improve park management.

The managers’ favorite aspects of plant management within the urban parks were related to maintenance (e.g., weed/invasive management, continuous improvement) and ecosystem management for functions and processes (i.e., in the natural‐passive use parks). Discussions about weed/invasive management were expected given the creation and implementation of the City of Portland's Invasive Plant Strategy to integrate invasive plant management into existing programs and reduce invasive plant coverage (City of Portland Bureau of Environmental Services 2008, 2010). Both the interview results and the ordination results (Figs. 1, 2) indicated that the managers of natural‐passive use parks most often discussed ecosystem management for functions and processes, which is the framework for Portland Parks and Recreation's “Natural Areas Restoration Plan Update” (Portland Parks and Recreation 2015b). The managers of recreational‐active use and multi‐use parks did not mention ecosystem management, and only one manager of a recreational‐active use park discussed Portland Parks and Recreation's Sustainable Landscapes Initiative, which is aimed at creating native habitat patches in developed parks within the city (Portland Parks and Recreation 2015a). This implies that park management initiatives within cities may not be adequately shared and emphasized in the work of different levels of management (e.g., senior level to “on‐the‐ground” managers and maintenance staff). While sustainable landscape initiatives can be beneficial, additional training and discussion of these initiatives throughout park administrations in Portland and other cities are necessary to have their intended positive outcomes on park ecosystem services.

Park visitors across all park types also described aspects that they liked about the vegetation such as trees, size, diversity, maintenance, and colors, and also shade and grass in recreational‐active use and multi‐use parks. Many of these aspects are consistent with previous research that shows various vegetation elements can have restorative effects on human health (van den Berg 2008). While this study was specific to Portland, Oregon, a moderately sized urban city in the USA with temperate climate and a range of urban park types, these vegetation preferences may be similar in comparable cities. However, vegetation preferences may differ across cities with more arid or semi‐arid climates, rural to suburban to urban land use, and lesser or more densely populated cities with varying demographics (Bjerke et al. 2006, Tilt 2010, Qiu et al. 2013, Hashim et al. 2016, Southon et al. 2017), and additional studies are needed to better understand how these preferences vary across park type in these settings. Most studies to date have also focused on urban areas in temperate climates, and so more studies should be completed across park types in arid and semiarid urban environments. Regardless of the setting, park managers should familiarize themselves with the vegetation preferences and concerns of park visitors and safeguard these attributes in conjunction with park ecological goals.

Interview responses showed a relatively high level of satisfaction among managers as well as visitors with the existing vegetation in Portland's parks. The most common response from managers was that they would make no changes, but some managers discussed improving maintenance (e.g., more pruning, weed management, etc.), increasing park staffing, adding more plants, updating infrastructure, and improving species selection. The most common response from the visitors was also to make no changes. However, other visitors indicated that they would like more color, followed by more flowers, invasive/harmful plant removal, more plants (i.e., middle growth/shrubs), and improved placement of plants. The ordination results show that visitor responses regarding plant choice and wanting more vegetation color were associated with multi‐use and recreational‐active use parks. There are opportunities to meet the preferences of both managers and visitors by continuing to manage trails/paths in natural‐passive use parks and removing weeds/invasive plants in all park types. Managers can also select a greater diversity of plants for recreational‐active use and multi‐use parks that have more color and flowers, but are also climate‐adapted, disease‐resistant, drought‐tolerant, and/or provide habitat for pollinators.

Park managers and park visitors described a variety of ways that they interpret accessibility in different types of urban parks. Park accessibility is important to examine because the benefits of urban greenspaces are not always shared equitably across different communities (Byrne 2012, Weems 2016, Frumkin et al. 2017). The managers we interviewed primarily described accessibility in terms of ADA/wheelchair access, trails/paths, and proximity. Visitors also viewed accessibility in terms of proximity and trails/paths for the natural‐passive use parks, but they also described maintenance (e.g., of vegetation and trails, cleanliness), access to relaxation opportunities, water, and benches. Visitors discussed ADA accessibility less often than the park managers. Some additional accessibility concerns that were not mentioned by the natural‐passive use park visitors, but were important to recreational‐active use and multi‐use park visitors, were if these parks had sufficient tree shade and open space, were kid and dog‐friendly, had water for recreation, and wheelchair accessibility. In this manner, the vegetation in each of the urban park types were important components of determining accessibility for many park visitors and should be maintained and/or enhanced by urban park managers. The results also indicate how perceptions of accessibility overlap somewhat, but are not entirely consistent across park manager and visitors. We recommend that park managers regularly solicit feedback and encourage dialogue with visitors about accessibility needs.

The ways in which managers perceived how visitors interacted with the vegetation and visitor comments appeared to influence many plant choices and/or design within the parks. When managers were asked how visitor perceptions of plants influenced plant choice and/or design in the parks, some of the most common answers were that managers attempted to eliminate hiding/camping places, improve aesthetics, and select hearty plants to withstand trampling. Park managers also indicated that they worked to remove hazardous plants based on comments they received from visitors, just as some visitors suggested when they were interviewed (Talal 2019). However, not all of the visitor feedback from our interviews in summer 2018 was represented in comments that visitors provided to park managers. This indicates a potential information gap and implies that managers should solicit additional visitor feedback about plant management in different parks. Additionally, many visitors in all park types also expressed a desire to learn more about the plants and animals in the parks. There are opportunities to provide more formal programming and interpretive signs/labels in multiple languages and with multisensory experiences that may better engage and educate park visitors (Kaplan et al. 1998, Byrne 2012).

While urban park managers often work toward meeting the preferences of park visitors, they also described various limitations that prevent them from managing parks in ways they might prefer. The most commonly mentioned limitation by park managers was funding, followed by limited staff resources and unfavorable visitor behaviors. This result is consistent with other research, which shows that managers often face funding limitations, depreciative behaviors by park visitors, and/or knowledge gaps related to cultural ecosystem services of urban parks (Budruk et al. 2003, Millennium Ecosystem Assessment 2005, Baur et al. 2013a, b, Chan et al. 2015, Svendsen et al. 2016, Palliwoda et al. 2017). Each park administration should create and/or continue to develop a strategic planning program that promotes visitor accessibility, meets the vegetation preferences of both park visitors and managers, and provides specific funding for the purchase and maintenance of urban park vegetation. In addition, continued development of interdisciplinary and mixed methods research has the potential to increase our understanding of vegetation preferences and improve overall management of urban green spaces. Overall, increased communication among government agencies, non‐profit organizations, and community members, as well as continued park investment have the potential to enhance the many social and ecological benefits of urban parks.
