# Supplementary material for: Vegetation management for urban park visitors: a mixed methods approach in Portland, Oregon
Source: Ecol Appl. 2020 Feb 24;30(4):e02079. doi: 10.1002/eap.2079 (PMC7317485; doi:10.1002/eap.2079)
Supplement: Supplementary file 1 [file EAP-30-e02079-s001.pdf]

**Supporting Information.** Talal, M.L., and M.V. Santelmann. 2020. Vegetation management for urban park visitors: a mixed methods approach in Portland, Oregon. Ecological Applications.

## Appendix S1. Park Visitor Demographics

**Table S1. Park visitor demographics information for Portland, all parks in the study, and by park type.**

| Park Type | Female (%) | Male (%) | Age Range | Age Median | Education (% of ≥ 25-years old with ≥ bachelor's degree) | White/ Caucasian (%) | Non-white minorities (%) |
|---|---|---|---|---|---|---|---|
| **Portland (United States Census Bureau 2017)** | 50.5 | 49.5 | -- | -- | 48 | 77 | 23 |
| **All Parks** | 53 | 47 | 19 – 75 | 37 | 57 | 72 | 28 |
| **Natural-passive use** | 38 | 62 | 19 – 75 | 37 | 83 | 92 | 8 |
| **Recreational-active use** | 67 | 33 | 19 – 71 | 37 | 60 | 67 | 33 |
| **Multi-use** | 53 | 47 | 25 - 64 | 35 | 33 | 60 | 40 |

## <u>Literature Citation</u>

United States Census Bureau. 2017. "QuickFacts Portland city, Oregon." Retrieved from https://www.census.gov/quickfacts/fact/table/portlandcityoregon/PST045217
