# Supplementary material for: Vegetation management for urban park visitors: a mixed methods approach in Portland, Oregon
Source: Ecol Appl. 2020 Feb 24;30(4):e02079. doi: 10.1002/eap.2079 (PMC7317485; doi:10.1002/eap.2079)
Supplement: Supplementary file 2 [file EAP-30-e02079-s002.pdf]

Supporting Information. Talal, M.L., and M.V. Santelmann. 2020. Vegetation management for urban park visitors: a mixed methods approach in Portland, Oregon. Ecological Applications.

Appendix S2. Institutional Review Board Instrument

Survey Questions: Perspectives of Urban Park Managers

1) How would you describe the look and feel of this park?

2) How often do you visit the park as part of your job and what do you do on-site?

3) What are the short and long-term management goals for this park?

4) What aspects, if any, would you change about management of this park?

5) What are the factors, if any, that limit your ability to manage the park in the way you might prefer?

6) What aspects, if any, do you like about how the plants are managed in this park?

7) What aspects, if any, would you like to change about the way plants are managed in this park?

8) Describe the typical visitors to this park. Who are they?

9) What range of activities do people typically do in this park?

10) Do you feel that this park is accessible to the needs of visitors?

11) In your opinion, how do park visitors interact with the plants in this park?

12) Do you think park visitors like the different types of?
    a. Trees:
    b. Shrubs/Saplings:
    c. Herbs:
    d. Vines (if applicable):

13) Do you think park visitors like the amount (number/cover) of?
    a. Trees:
    b. Shrubs/Saplings:
    c. Herbs:
    d. Vines (if applicable):

14) Have you received comments from park visitors about the management of this park? If so, describe.

15) Have you received comments from park visitors about the plants in this park? If so, describe.

16) How do park visitor experiences influence this park's management (previously and/or currently)?

17) Specifically, how does park visitor experience influence vegetation choice and/or design in this park?

18) Have you observed or heard about any equity issues in the parks you manage? If so, how are you addressing these issues?

19) Background Questions: How long have you worked at Portland Parks and Recreation? How many years have you worked in your profession?

20) Would you like to share any additional comments about this park?

Perspectives of Urban Park Managers: Verbal Consent Guide

Purpose: The purpose of this project is to explore the perspectives of urban parks in Portland and how visitor perceptions and experiences with the vegetation may influence park management by implementing qualitative research methods (semi-structured interviews). The results of this project may be used to provide information on park management goals, park visitor experiences, how park visitor experiences and perceptions may influence park management, and potential areas for improvement in urban parks. In order to be in this study, you must be of legal age to consent, which is 18 in most states.

Activities: Park managers will be asked a series of questions about their park management experiences, as well as some background questions.

Risks: Managers may have some discomfort sharing their park management experiences, but they can stop the interview at any time. Any information that is volunteered by participants that discloses illegal activity will not be collected or recorded.

Payment: There will be no payment for the interview.

Confidentiality: No information will be collected regarding the subjects' identity, and complete confidentiality will be maintained. Unidentifiable data will be shared with the Urban Water Innovation Network.

Voluntariness: Participation in the study is voluntary and there is no penalty for choosing to no participate in the study or leave at any time. Interviewees are free to skip any questions.

Contact Information:

Principal Investigator: Dr. Mary Santelmann, Oregon State University, santelmm@oregonstate.edu

Student Investigator: Michelle Talal, Oregon State University, talalm@oregonstate.edu

Sponsor: National Science Foundation Grant #1444758: Urban Water Innovation Network (UWIN): Transitioning Toward Sustainable Urban Water Systems
