# Supplementary material for: Vegetation management for urban park visitors: a mixed methods approach in Portland, Oregon
Source: Ecol Appl. 2020 Feb 24;30(4):e02079. doi: 10.1002/eap.2079 (PMC7317485; doi:10.1002/eap.2079)
Supplement: Supplementary file 3 [file EAP-30-e02079-s003.pdf]

Supporting Information. Talal, M.L., and M.V. Santelmann. 2020. Vegetation management for urban park visitors: a mixed methods approach in Portland, Oregon. Ecological Applications.

Appendix S3. Park Manager Interview Codebook

General Notes: Coding in NVivo will only be completed for the answers of seven questions asked during the interviews. These include the following:

A) What are the factors, if any, that limit your ability to manage the park in the way you might prefer?
B) What aspects, if any, do you like about how the plants are managed in this park?
C) What aspects, if any, would you like to change about the way plants are managed in this park?
D) Do you feel that this park is accessible to the needs of visitors?
E) In your opinion, how do park visitors interact with the plants in this park?
F) Have you received comments from park visitors about the plants in this park? If so, describe.
G) Specifically, how does park visitor experience influence vegetation choice and/or design in this park?

PROTOCOL:

1) **Do not code interview questions. Only code answers from participants.**
2) **All statements should have at least a 1st order code and a 2nd order code. Some statements might have more than one 2nd order code. Some statements have a 1st, 2nd, and 3rd order codes.**
   EXAMPLES:
   - "*A limitation is our lack of funding*" (1st order code: Limitations, 2nd order code: funding)
   - "*Limitations include our lack of funding and staff resources*" (1st order code: Limitations, 2nd order code: Funding, budget, 2nd order code: Staff resources).
   - "*Limitations include our lack of funding and visitors who throw trash* (1st order code: Limitations, 2nd order code: Funding, budget, 2rd order code: Unfavorable visitor behaviors, 3rd order code: Trash).
3) **Code the entire statement provided by an individual in the same paragraph even if there are multiple codes. This is useful for visualizing the complexity of participant responses during the analysis.**
   EXAMPLE:
   - *"Limitations include our lack of funding and staff resources. We also have some issues with enforcement and people throwing trash"* (1st order code: Limitations, 2nd order code: Funding, budget, 2nd order code: Staff resources, 2nd order code: enforcement, 2nd order code: Unfavorable visitor behaviors, 3rd order code: Trash).

1ST ORDER CODES: Note: 1st order codes are the entire responses of participants for each question:

- **"A – LIMITATIONS"** – Response for "what are the factors, if any, that limit your ability to manage the park in the way you might prefer?"
- **"B – WHAT LIKE ABOUT HOW PLANTS ARE MANAGED"** -- Response for "what aspects, if any, do you like about how the plants are managed in this park?"
- **"C – WOULD LIKE TO CHANGE ABOUT PLANT MANAGEMENT"** – Response for "What aspects, if any, would you like to change about the way plants are managed in this park?
- **"D – ACCESSIBILITY"** – Response for "do you feel that this park is accessible to the needs of visitors?"
- **"E – HOW VISITORS INTERACT WITH PLANTS"** – Response for "in your opinion, how do park visitors interact with the plants in this park?"
- **"F – COMMENTS FROM VISITORS ABOUT PLANTS**" – Response for "have you received comments from park visitors about the plants in this park? If so, describe."
- **"G – HOW VISITOR PERCEPTION OF PLANTS INFLUENCES PLANT CHOICE OR DESIGN"** – Response for "specifically, how does park visitor experience influence vegetation choice and/or design in this park?"

2nd ORDER CODES FOR "A – LIMITATIONS"

- Coordination in parks department
- Design Plan
- Enforcement
- Equipment, supplies
- Funding, budget
- Information (scientific), more
- Infrastructure, needs improvement
- Safety, terrain
- Staff resources
- Unfavorable visitor behaviors
  - **3rd ORDER CODES:**
    - Dogs off-leash
    - Heavy usage, crowding
    - Homelessness, camping
    - Trash
- Vegetation
- Water use, limited

2nd ORDER CODES FOR "B – WHAT LIKE ABOUT HOW PLANTS ARE MANAGED"

- Beauty

- Color, seasonal
- Design, layout
    - **3rd ORDER CODES**
        - Like design, layout
        - Problematic design, layout
- Different crews in parks department specialize
- Ecosystem management, functions and processes: include ecological condition
- Grass
- Habitat variation
- Horticultural beds
- I don’t like how plants are managed
- Maintenance
    - **3rd ORDER CODES**
        - Irrigation
        - Low maintenance
        - Pruning
        - Weed, invasive management
        - Well-maintained, improvement continuous
- Maturity, age of plants
- Native species
- Naturalistic
- Plant variety
- Shade
- Trees
- Understory
- Volunteers

2nd ORDER CODES FOR “C – WOULD LIKE TO CHANGE ABOUT PLANT MANAGEMENT”

- Design, new
- Funding, budget, more
- Infrastructure, update
- Maintenance, improve
    - Fertilize, more
    - Pruning, more
    - Water, more
    - Weed management, more
- No changes
- Placement, improve
- Plants, more

- **3rd ORDER CODES**
  - Midstory, shrubs more
  - Plantings, generally more
  - Trees, more
  - Understory, more
- Species selection, improve
  - **3rd ORDER CODES**
    - Climate adaptable
    - Disease resistant
    - Diversity
    - Drought tolerant
    - Herbaceous perennials
- Staffing, more
- Training for staff, more
- Unfavorable visitor behavior, less
  - **3rd ORDER CODES**
    - Visitor damage, less
    - Homeless camps, reduce

2nd ORDER CODES FOR "D – ACCESSIBILITY"

- Accessible general: include: yes, no requests, no additional detail
- ADA, wheelchair accessibility
  - **3rd ORDER CODES**
    - Not entirely, could improve ADA accessibility
    - Yes, ADA accessible
- Boat ramp
- Clean
- Design, open
- Grass, turf
- Not sure, not developed
- Parking
- Playground
- Proximity, location, local access
- Signs, mostly in English only
- Size, could be bigger
- Trails/Paths
  - **3rd ORDER CODES**
    - Yes, paths, trails accessible
    - Not entirely accessible paths, trails, only to some
- Water, drinking

- Welcoming atmosphere
- Wildlife and nature access

2nd ORDER CODES FOR "E – HOW VISITORS INTERACT WITH PLANTS"

- Access, hazard issues with plants
- Environmental education
- Grass, turf
- Not a lot of interaction
- Play in plants
- Research
- Respectful appreciation of plants
- Shade, primarily from trees
- Stewardship, volunteers
- Unfavorable visitor behaviors
    - **3rd ORDER CODES**
        - Camping, hiding in shrubs
        - Dog off-leash
        - Garbage, throwing
        - Graffiti
        - Picking flowers
        - Trampling, breaking
- Viewing, visual
    - **3rd ORDER CODES**
        - Beauty
        - Birds and habitat
        - Color
        - Flowers
        - Garden
        - Seasonal
        - Trees
        - Variety of plants
        - Visual, general
- Walk around plants

2nd ORDER CODES FOR "F – COMMENTS FROM VISITORS ABOUT PLANTS"

- Diversity of shrubs, want more
- Graffiti on trees
- Hazard vegetation
- Invasive plants
- No comments from visitors

- Plant removal concern
- Plantings, new
- Replacement request (dead or broken)
- Visitors like the vegetation

2nd ORDER CODES FOR "G – HOW VISITOR PERCEPTION OF PLANTS INFLUENCES PLANT CHOICE OR DESIGN"

- Aesthetics, improvements
- Balance ecological and human needs
- Design, changed
- Design, kept
- Eliminate hiding, camping places
- Hazard plant removal
- Hearty plants, to withstand trampling
- Not a lot of public, visitor input
- Plantings to hide residences
- Plants add in popular areas
- Plants to guide human traffic
- Public input during planning
- Trail access
- Views, maintain
- Volunteer stewardship involvement
