# Supplementary material for: Vegetation management for urban park visitors: a mixed methods approach in Portland, Oregon
Source: Ecol Appl. 2020 Feb 24;30(4):e02079. doi: 10.1002/eap.2079 (PMC7317485; doi:10.1002/eap.2079)

Supporting Information. Talal, M.L., and M.V. Santelmann. 2020. Vegetation management for urban park visitors: a mixed methods approach in Portland, Oregon. Ecological Applications.

Appendix S4. Results Summary Figures

Figure S1. Attributes that managers commonly liked about how the plants are managed in all parks, and by park type (n = 21 interviews).

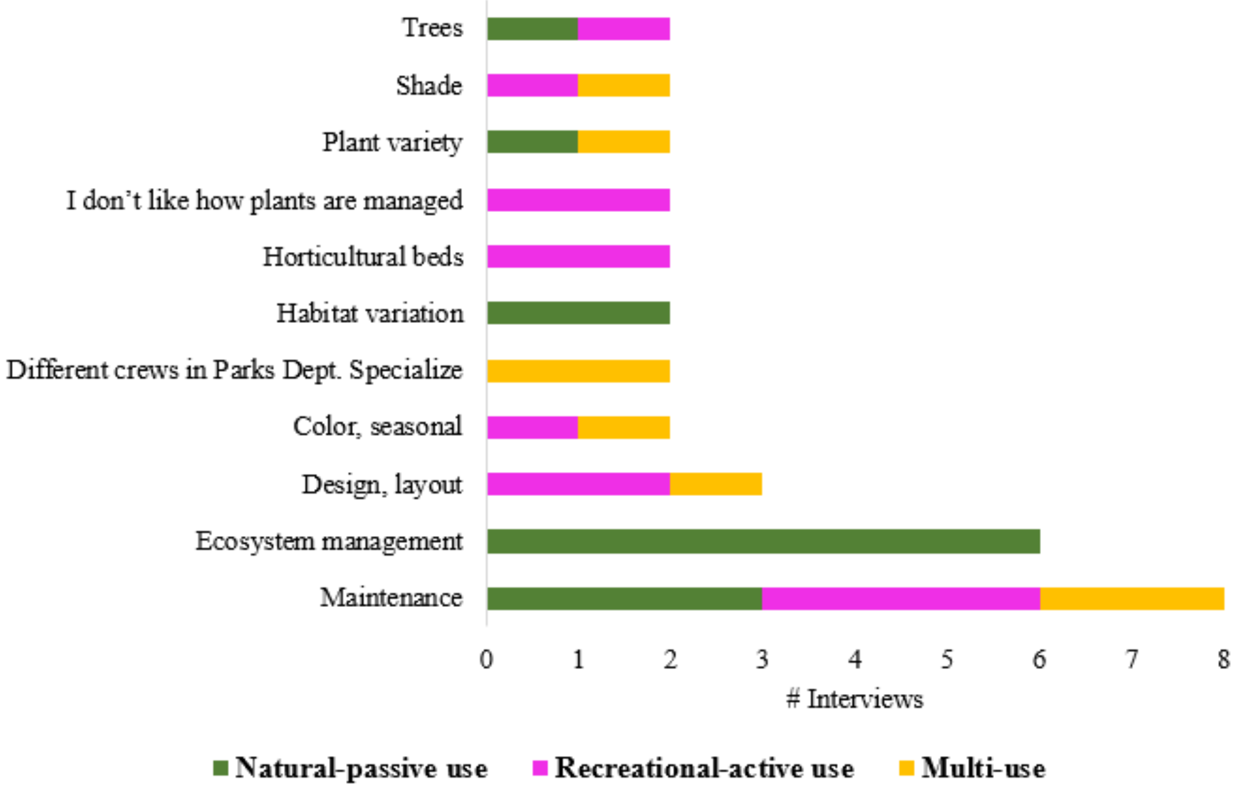

Figure S2. Attributes that managers commonly would like to change about how the plants are managed in all parks, and by park type (n = 21 interviews).

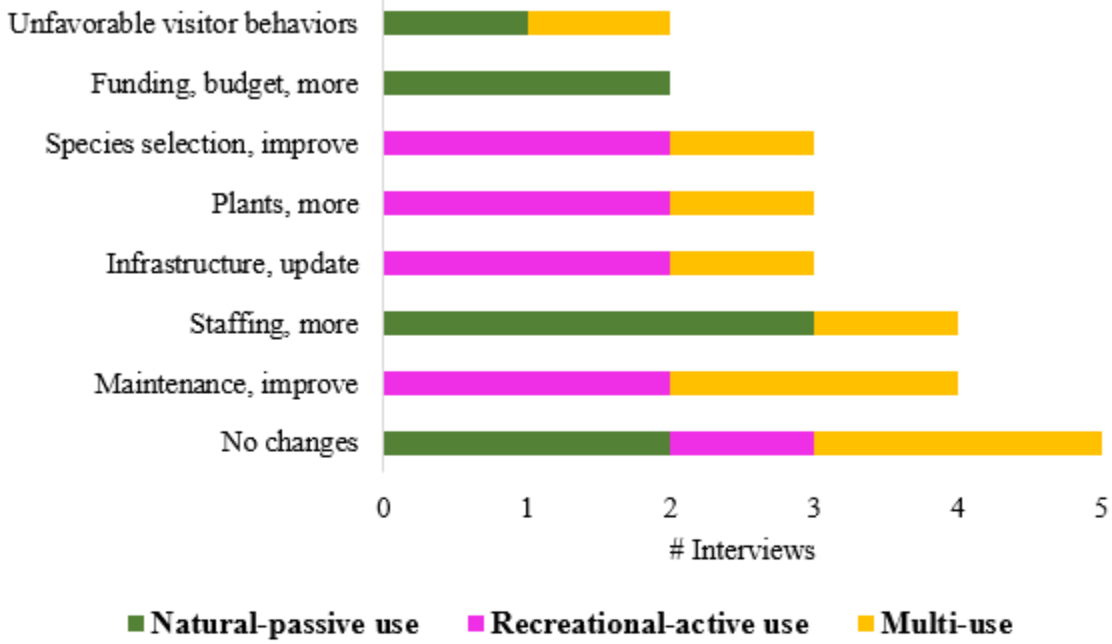

Figure S3. Visitor accessibility concerns commonly perceived by managers in all of the parks, and by park type (n = 21 interviews).

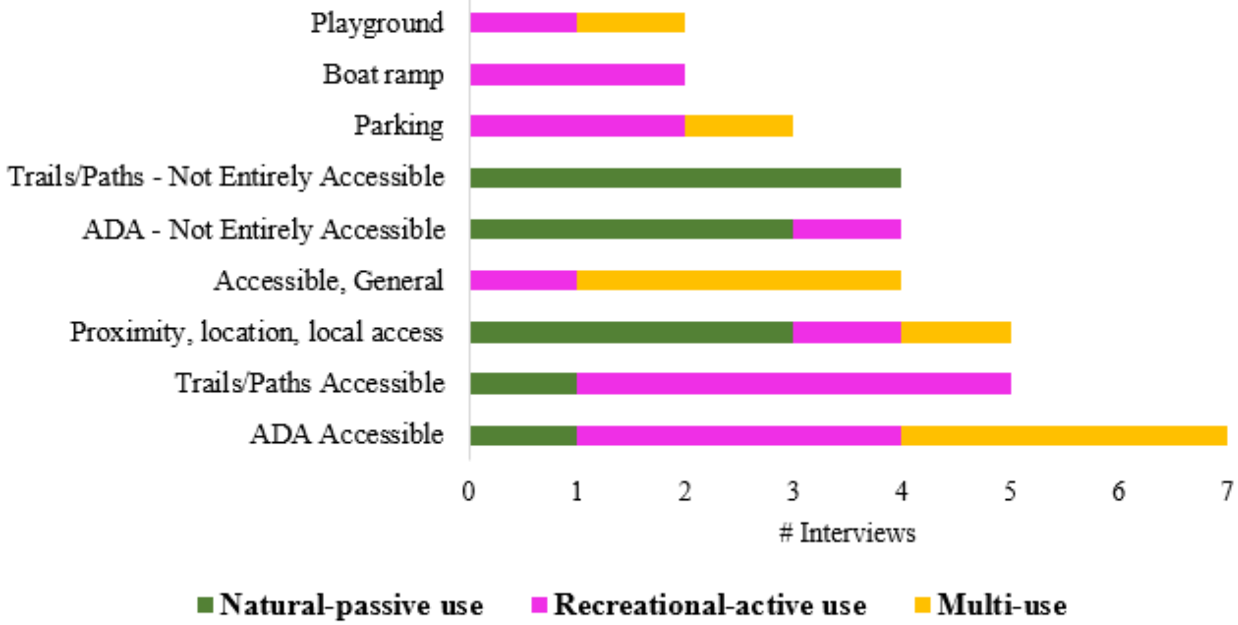

Figure S4. Comments that managers commonly receive from visitors about plants in all of the parks, and by park type (n = 21 interviews).

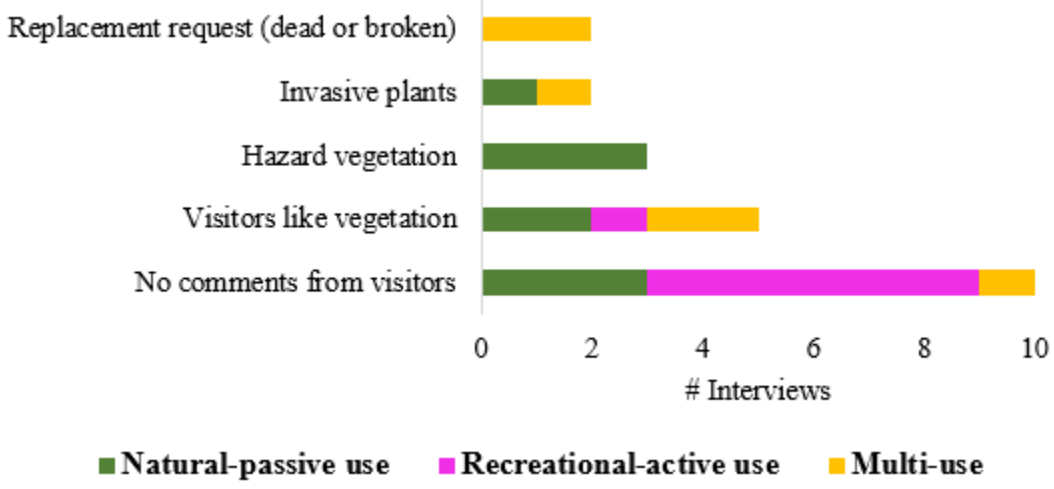

Figure S5. Common ways that visitor perception of plants influences plant choice and/or design in all parks and by park type (n = 21 interviews).

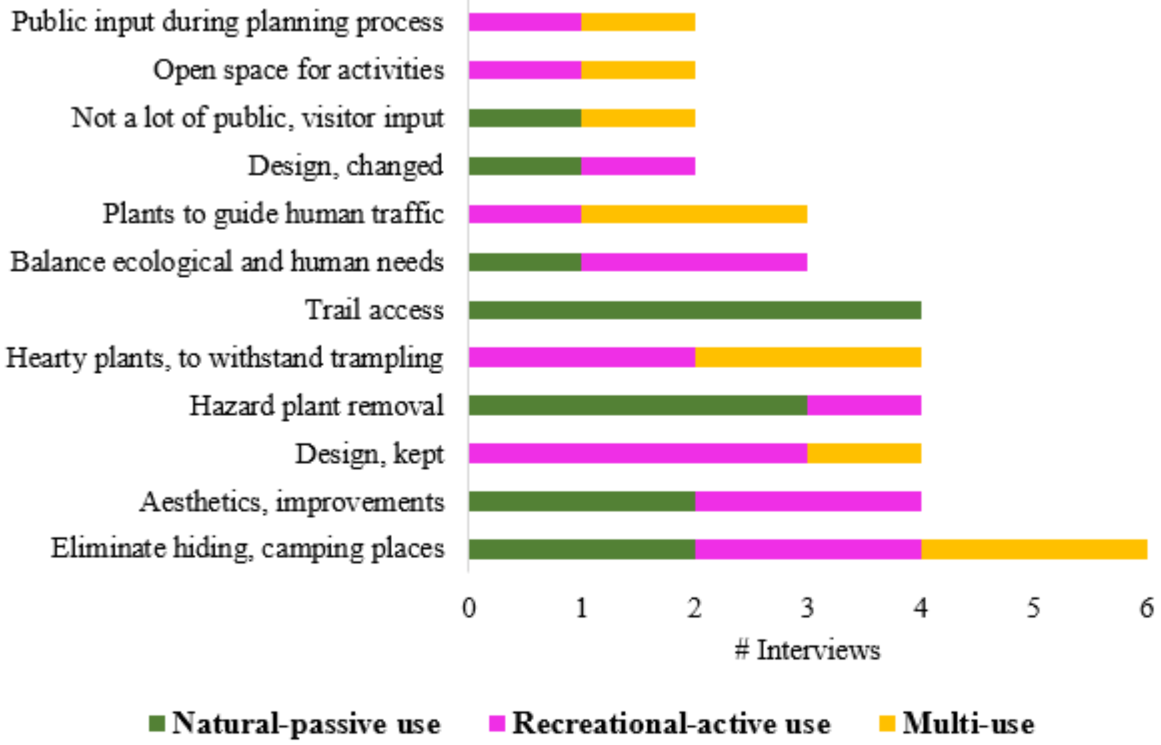

Figure S6. Common limitations to managing the parks in the way the managers might prefer for all parks, and by park type (n = 21 interviews).

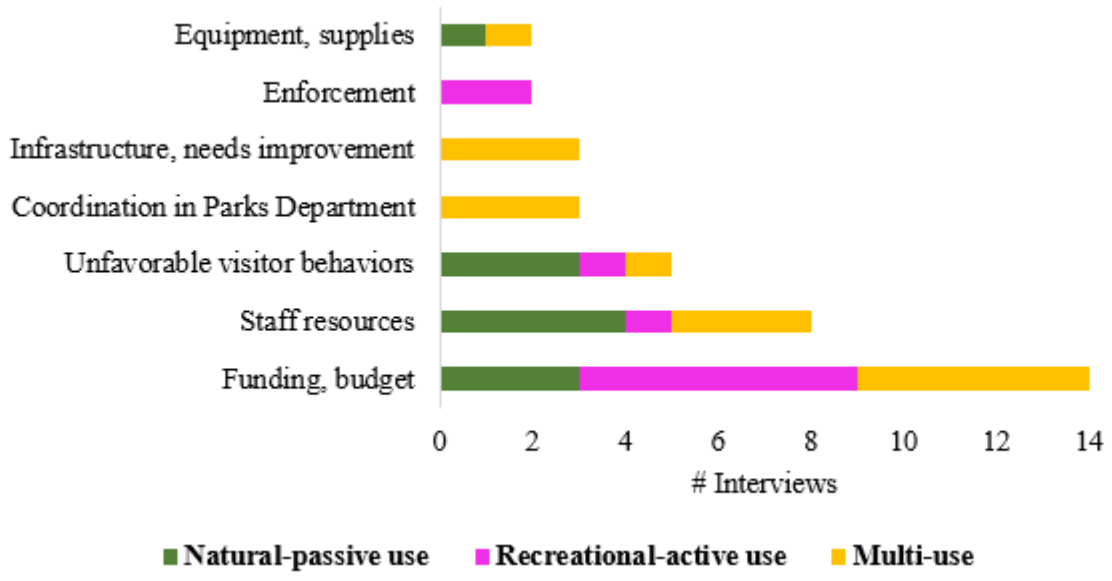

Supplement: Supplementary file 4 [file EAP-30-e02079-s004.pdf]
