# Supplementary material for: Vegetation management for urban park visitors: a mixed methods approach in Portland, Oregon
Source: Ecol Appl. 2020 Feb 24;30(4):e02079. doi: 10.1002/eap.2079 (PMC7317485; doi:10.1002/eap.2079)
Supplement: Supplementary file 5 [file EAP-30-e02079-s005.pdf]

Talal, M.L., and M.V. Santelmann. 2020. Vegetation management for urban park visitors: a mixed methods approach in Portland, Oregon. Ecological Applications.

Data S1

Park Manager and Visitor Interview Count Data and Plant Community Composition Data

---

Authors

Michelle L. Talal
Oregon State University
Graduate School
2900 SW Jefferson Way
Corvallis, OR 97331
michelle.talal@gmail.com

Mary V. Santelmann
Oregon State University
College of Earth Ocean and Atmospheric Sciences
104 CEOAS Administration Building
Corvallis, OR 97331
mary.santelmann@oregonstate.edu

File list and description

The Data S1 File contains park manager count data (collected 2018 – 2019), park visitor interview count data (collected summer 2018), and plant community and composition data (collected summer 2017) for 15 urban parks in Portland, Oregon USA. For more detail on the definitions of the interview response variables, please see Appendix S3 for the codebook.

`DataS1 Manager Responses – All Interviews` – this file contains point count data for all the park manager interviews, including what they liked about how the plants are managed in their park, what they would like to change about plant management in their park, accessibility, how they perceive visitors to interact with the plants in their park, comments from visitors about the plants, how visitor perception includes plant choice or design, and limitations. Each point represents an individual park manager who stated a particular opinion. For more information on the meanings of the responses, please see

Appendix S3 for the codebook. The number of responses is shown for all parks and also divided by park type (natural-passive use, recreational-active use, and multi-use).

`DataS1 Manager Responses at Least 10% Interviews` – this file contains point count data for responses provided in at least 10 percent of the park manager interviews, including what they liked about how the plants are managed in their park, what they would like to change about plant management in their park, accessibility, how they perceive visitors to interact with the plants in their park, comments from visitors about the plants, how visitor perception includes plant choice or design, and limitations. Each point represents an individual park manager who stated a particular opinion. For more information on the meanings of the responses, please see Appendix S3 for the codebook. The number of responses is shown for all parks and also divided by park type (natural-passive use, recreational-active use, and multi-use).

`DataS1 Vegetation Likes and Dislikes – Visitors and Managers` – this file contains point count data for visitor and manager responses about what they liked about how the plants in each park. For more information on the meanings of the responses, please see Appendix S3 for the codebook.

`DataS1 Vegetation Dislikes – Visitors and Managers` – this file contains point count data for visitor and manager responses about what they disliked and would like to change about how the plants in each park. For more information on the meanings of the responses, please see Appendix S3 for the codebook.

`DataS1 Accessibility - Visitors and Managers` – this file contains point count data for visitor and manager responses about park accessibility in each park. For more information on the meanings of the responses, please see Appendix S3 for the codebook.

`DataS1 How Visitors Influence Plant Mgmt` – this file contains point count data for manager perspectives on how visitors influence plant management in each park. For more information on the meanings of the responses, please see Appendix S3 for the codebook.

`DataS1 Comments from Visitors to Managers and Manager Limitations` – this file contains point count data comments that managers indicated they had received from park visitors about the vegetation in each of the parks. The file also contains point count data for what the managers indicated were their limitations to managing their parks in the ways that they might prefer. For more information on the meanings of the responses, please see Appendix S3 for the codebook.

`DataS1 Park Sample Units and Percent Cover` – this file contains plant community composition percent cover data for each of the park sample units (averaged for five 400m^2 sample plots for each park sample unit).

DataS1 Plant Species Notes- this file contains plant species codes and scientific names for each of the plants identified within the park sample plots.
